# Xiao Chai Hu Tang-derived decoction (Tung-Yi Fang) suppresses triple negative breast cancer cells *in vitro* and *in vivo* via regulating EGFR/AXL-mediated signaling

**DOI:** 10.3389/fphar.2026.1778030

**Published:** 2026-03-13

**Authors:** Li-Lan Liao, Zhi-Hu Lin, Chia-Ching Liaw, Hsin Yeh, Wei-Hao Wang, Yun-Chih Chen, Yi-An Lin, Ai-Jung Tseng, Yu-Chun Lin, Wen-Hsin Tsai, Chi-Hong Chao, Mei-Kuang Lu, Chung-Hua Hsu, Tung-Yi Lin

**Affiliations:** 1 Institute of Traditional Medicine, National Yang Ming Chiao Tung University, Taipei, Taiwan; 2 Branch of Linsen Chinese and Kunming, Taipei City Hospital, Taipei, Taiwan; 3 Traditional Chinese Medicine Glycomics Research Center, National Yang Ming Chiao Tung University, Taipei, Taiwan; 4 School of Chinese Medicine, National Yang Ming Chiao Tung University, Taipei, Taiwan; 5 National Research Institute of Chinese Medicine, Ministry of Health and Welfare, Taipei, Taiwan; 6 Department of Pharmacy, National Yang Ming Chiao Tung University, Taipei, Taiwan; 7 School of Chinese Medicine for Post-Baccalaureate, I-Shou University, Kaohsiung, Taiwan; 8 Department of Biological Science and Technology, National Yang Ming Chiao Tung University, Hsinchu, Taiwan; 9 Center For Intelligent Drug Systems, National Yang Ming Chiao Tung University, Hsinchu, Taiwan

**Keywords:** anti-cancer, herbal formula, triple-negative breast cancer, tyrosine kinase receptor, XCHT-derived Tung-Yi Fang

## Abstract

**Background:**

Triple-negative breast cancer (TNBC) is an aggressive breast cancer subtype with limited therapeutic options. Xiao Chai Hu Tang (XCHT), a classical herbal formula, is prescribed as an adjuvant therapy in breast cancer care. However, the high sugar content of XCHT may influence its anticancer efficacy under modern experimental conditions. This study investigated the anti-TNBC effects of Tung-Yi Fang (TYF), a sugar-reduced XCHT-derived decoction, and elucidated its underlying mechanisms *in vitro* and *in vivo*.

**Methods:**

TYF was chemically characterized by reverse-phase high-performance liquid chromatography. Its bioactivities were evaluated in TNBC cells using viability and colony formation assays, as well as migration/invasion, cell cycle, and apoptosis analyses. Mechanistic insights were investigated using receptor tyrosine kinase (RTK) arrays, Western blotting, and immunofluorescence analyses. The *in vivo* relevance of TYF was further assessed in an orthotopic 4T1-luciferase breast cancer mouse model.

**Results:**

TYF selectively suppressed TNBC cell growth while exerting minimal effects on non-tumorigenic breast epithelial cells and fibroblasts. TYF induced G2/M cell cycle arrest and apoptotic responses. TYF impaired TNBC cell mobility through disruption of actin cytoskeletal organization and suppression of FAK/Src signaling. RTK profiling and downstream analyses revealed that TYF concurrently regulated EGFR- and AXL-associated signaling pathways, leading to attenuation of AKT, ERK, and STAT3 activation. TYF administration significantly reduced tumor growth and metastatic burden without detectable systemic toxicity *in vivo*.

**Conclusion:**

TYF exhibits anti-TNBC activity through EGFR and AXL inhibition and suppression of metastatic potential. Its efficacy highlights the therapeutic potential of glucose-controlled herbal formulations in cancer management.

## Introduction

Breast cancer represents a biologically heterogeneous malignancy encompassing multiple molecular subtypes with distinct pathological features, clinical behaviors, and therapeutic responses (Fragomeni et al., 2018; Wang and Wu, 2023). Among these subtypes, triple-negative breast cancer (TNBC), defined by the absence of estrogen receptor (ER), progesterone receptor (PR), and HER2 expression, accounts for approximately 15%–20% of all breast cancer cases (Almansour, 2022). TNBC is frequently associated with aggressive tumor growth, early metastatic dissemination, and limited treatment options due to the lack of actionable molecular targets (Dent et al., 2007). Despite advances in chemotherapy and emerging immunotherapeutic approaches, disease recurrence and poor prognosis remain common (Bonotto et al., 2014), underscoring the need to explore complementary strategies that address the complex biological nature of TNBC.

Currently, integrative medical approaches have attracted increasing attention in the supportive management of breast cancer. A retrospective clinical observational study suggests that for stage IV advanced breast cancer, the 5-year average survival rate is higher in patients who take traditional Chinese medicine (TCM). Compared to those who do not take traditional Chinese herbal medicine, the risk of death is reduced (hazard ratio, [HR]: 0.45) (Chen et al., 2023). Similarly, another meta-analysis of breast cancer showed that the overall survival rate of breast cancer patients treated with Chinese herbal medicine was significantly higher than that of those who did not receive treatment (Ho et al., 2021). These findings indicated that TCM may contribute to beneficial outcomes of patients with advanced breast cancer.

Xiao Chai Hu Tang (XCHT), recorded in the *Shang Han Lun* of the Han Dynasty, is a classic formula specifically used for treating Shaoyang syndrome. Specifically, in the TCM theory, XCHT may have therapeutic effects for diseases caused by emotional disturbances and Qi stagnation in the liver and gallbladder meridians, which provide an ethnopharmacological rationale for the frequent clinical use of XCHT-related prescriptions in breast cancer management (Hempen and Fischer, 2009; Li et al., 2021). Some studies showed that XCHT exhibits therapeutic effects on emotional disorders and inflammatory diseases, such as depression, pancreatic fibrosis, and cancer, caused by dysfunction in the liver and gallbladder meridians (Zhang et al., 2023; Zhang et al., 2015). For example, XCHT alleviates the progression of liver cancer by inhibiting the activation of hepatic stellate cells and regulating mitochondria-mediated apoptotic responses (Sakaida et al., 1998; Zhao et al., 2017). XCHT regulates the TLR4-mediated signaling pathway via gut microbiota modulation, thereby inhibiting tumor growth and improving depressive-like symptoms associated with liver cancer (Shao et al., 2021). Moreover, XCHT is the second commonly used TCM formula for treating liver cancer in Taiwan (Ting et al., 2017). Interestingly, a study showed the relationship between liver and gallbladder meridians-mediated emotional balance and the occurrence of breast cancer and indicated that XCHT has high potential for treating TNBC (Ho et al., 2021; Zheng et al., 2022).

**SCHEME 1:**
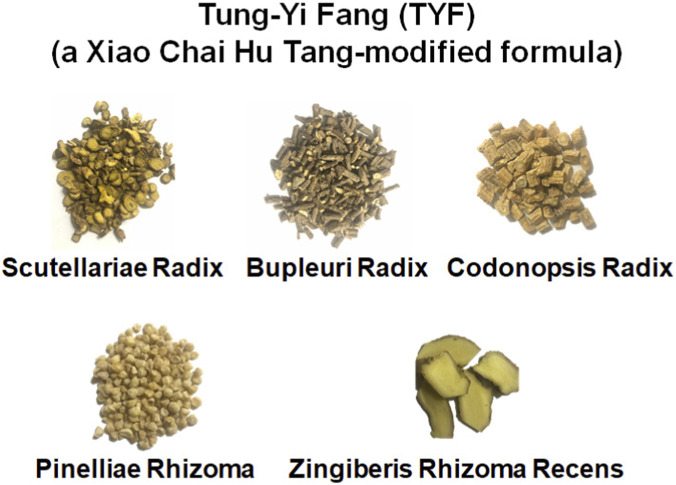
Compositions of TYF.

XCHT is composed of seven medicinal herbs in specific proportions, including Huangqin (Scutellariae Radix; root of *Scutellaria baicalensis* Georgi), Chaihu (Bupleuri Radix; root of *Bupleurum chinense* DC.), Dangshen (Codonopsis Radix; root of *Codonopsis pilosula* (Franch.) Nannf.), Banxia (Pinelliae Rhizoma; tuber of *Pinellia ternate* (Thumb.) Makino), Shengjiang (Zingiberis Rhizoma Recens; rhizomes of *Zingiber officinale* Roscoe), ZhiGancao (Glycyrrhizae Radix et Rhizoma; rhizomes of *Glycyrrhiza uralensis* Fisch.), and Dazao (Jujubae Fructus; fruit of *Ziziphus jujuba* Mill.). Although XCHT has been reported to have great effects for patients with breast cancer, there are no clinical reports demonstrating its effectiveness in blocking tumor progression or metastasis in patients with TNBC. Interestingly, a study showed that high carbohydrate intake increases the risk of breast cancer (odds ratio, [OR] = 1.76) (Sasanfar et al., 2021). Moreover, glucose metabolic reprogramming contributes to the progression and drug resistance of TNBC (Lei et al., 2023). The sugar-rich ingredients, ZhiGancao and Dazao, were thus removed as a methodological refinement to minimize metabolic confounding when evaluating XCHT-related biological relevance in TNBC models. Based on the TCM theory, the resulting XCHT-derived decoction was designated Tung-Yi Fang (TYF, Scheme 1).

In this study, we systematically examined the chemical profile and biological effects of TYF using *in vitro* TNBC models and an orthotopic breast cancer mouse model to clarify the biological relevance of XCHT-related prescriptions in TNBC-associated disease states. We further explored signaling pathways associated with tumor growth and metastatic behavior, with particular focus on EGFR- and AXL-related networks. Through this ethnopharmacological and mechanistic approach, we aimed to provide biological insight into the traditional use of XCHT-derived prescriptions in managing complex disease states associated with triple-negative breast cancer.

## Materials and methods

### Preparation of the water-soluble TYF

The ingredients of TYF, including Huangqin (Scutellariae Radix; *S. baicalensis* Georgi, batch number: 001202A104), Chaihu (Bupleuri Radix; *B. chinense* DC., batch number: 000902A10), Dangshen (Codonopsis Radix; *C. pilosula* (Franch.) Nannf., batch number: 002002A101), Banxia (Pinelliae Rhizoma; *Pinellia ternata* (Thunb.) Makino, batch number: 0005041A101) were purchased from Keda Pharmaceutical Co., Ltd. (Tainan, Taiwan). Shengjiang (Zingiberis Rhizoma Recens; Zingiber officinale Roscoe) was purchased from Hsinchu Production Area, Taipei Farmers’ Association Distribution Center. To prepare the water-soluble XCHT and TYF solution, add 20 times the amount of water to the herbal materials, bring to a boil over high heat, then simmer for about 30 min. After removing the herbal residues, approximately 270 mL remains (adjusted according to the standard prescription published by the Ministry of Health). After filtering the decoction, centrifuge at 25 °C, 2000 × g for 5 min, and distribute into 50 mL tubes (25 mL/tube), which are stored in a −80 °C freezer. The following day, remove the tubes and freeze-dry at −60 °C and 0.2 torr (KINGMECH) for approximately 4–5 days. The concentrated powder of water-soluble TYF was dissolved in PBS and 25% DMSO solution to form a concentration of 200 mg/mL. The TYF solution was stored in a −20 °C freezer for future use.

### Chemical characterization of TYF

The chemical fingerprint of TYF was characterized using reverse-phase high-performance liquid chromatography (RP-HPLC) equipped with a diode-array detector (DAD). Chromatographic separation was carried out on a SHIMADZU Nexera-*i* LC-2050C 3D system (Kyoto, Japan), employing a COSMOSIL 5C_18_-AR-II ODS column (ID 4.6 mm × 250 mm, particle size 5 µm). The analytical procedure was performed as follows: (1) TYF powder (10 mg) was dissolved in 1.0 mL of deionized distilled water (DDW). The solution is filtered through a nylon filter (0.22 μm, Syringe Filter Nylon) and 3 μL of the filtrated sample is injected into the HPLC system for analysis. (2) The mobile phase consists of solvent I: DDW containing 0.3% phosphoric acid (PA) and solvent II: acetonitrile (ACN) supplemented with 0.3% PA, with a programmed gradient elution as follows: 0–5 min, 0–3% ACN; 5–15 min, 3–15% ACN; 15–33 min, 15–20%ACN; 33–34 min, 20–23% ACN; 34–50 min, 23–30% ACN; 50–65 min, 30–50% ACN; 65–75 min, 50–100% ACN. (3) The standard solution of commercial compounds was prepared by dissolving 1.0 mg in 1.0 mL of methanol (MeOH). Each of 1, 2, 5, 8, and 10 μL were injected to construct a calibration curve. (4) Chromatographic conditions were set as follows: UV detection at 210 nm, flow rate of 1.0 mL/min, and a column temperature maintained at 40 °C. Retention time and peak area of each analyte were recorded. Quantification was achieved by comparing the test sample data with the calibration curve, enabling the determination of the concentration of the components in the TYF sample.

### Cell lines and cell culture

MDA-MB-231 cells and 4T1 cells were maintained as previously described (Hsu et al., 2013). WI-38 cells were cultured in Minimum Essential Medium (MEM; GIBCO-Life Technologies, United States) containing 10% fetal bovine serum (FBS; Gibco), 2 mM L-glutamine, and 0.1 mM non-essential amino acids (NEAA). MCF-10A and MCF-12A cells were grown in DMEM/F12 (Dulbecco’s Modified Eagle Medium: Nutrient Mixture F-12; GIBCO-Life Technologies, United States) supplemented with 5% horse serum, human epidermal growth factor (EGF, 20 ng/mL)/insulin (10 μg/mL), and hydrocortisone (500 ng/mL). All cell lines were incubated at 37 °C in a humidified atmosphere with 5% CO_2_. Cells were routinely passaged, and experiments were performed when cultures reached approximately 80% confluence.

### Cell viability assay

The cytotoxic effects of TYF on TNBC were evaluated using a crystal violet-based viability assay (Lin et al., 2023). Cells (5 × 10^4^ cells per well) were seeded in 12-well plates and incubated until firm attachment was achieved. Cells were subsequently exposed to TYF (0–800 μg/mL) for continuous treatment periods of 24–72 h. At the end of treatment, culture media were aspirated, and cells were fixed with 70% methanol for 10 min. Fixed cells were then stained with crystal violet solution for 1 h at room temperature. To solubilize the bound dye, 1 mL of 30% acetic acid was added to each well. An aliquot of the resulting solution (100 μL per well) was transferred to a 96-well plate, and absorbance was measured at 570 nm using a TECAN Magellan microplate reader (Thermo Scientific, United States).

### Colony formation assay

The clonogenic potential of TNBC cells following TYF treatment were assessed using a colony formation assay (Hua W‐J. et al., 2023). MDA-MB-231 and 4T1 cells were seeded in 6-well plates at densities of 500 cells per well and 1000 cells per well, respectively. Cells were allowed to grow under standard culture conditions until visible colonies were formed, typically within 4–7 days. Cells were then treated with TYF (200 μg/mL) for 3 consecutive days. Following treatment, cells were fixed and stained with 0.1% crystal violet solution containing 10% methanol for 1 h. Excess staining solution was removed by gentle washing, and plates were air-dried. Colonies were subsequently visualized and counted under a light microscope.

### Wound healing assays

MDA-MB-231 cells were seeded as a monolayer in a 12-well plate and cultured in an incubator until the cells were attached. After cell attachment, the culture medium was replaced with serum-reduced medium (0.5% FBS) and cells were incubated overnight to minimize the influence of proliferation on migration. The following day, a straight-line scratch was made in the wells using a 10 μL tip to simulate a wound. Then, cells were treated with TYF (0–400 μg/mL) to observe the extent of wound healing. The wound healing was observed and recorded at 0, 12, and 24 h using a phase-contrast microscope. The extent of wound healing, reflecting the difference in cell migratory ability, was then calculated using GraphPad Prism 8.

### Transwell assay

This study used the Transwell® assay (Lin et al., 2019) to assess the effects of TYF on the migration and invasion abilities of TNBC cells. A cell culture chamber (Corning, United States) was used and placed in a 24-well plate pre-filled with 1 mL of culture medium containing 10% FBS (1 mL/well). For the invasion assay, 30 µL of basement membrane matrix (Becton Dickinson Biosciences, United States) was first applied to the membrane of the cell culture chamber and incubated in the incubator for 30 min. For both the migration and invasion assays, the following steps were the same. A cell suspension containing 5 × 10^4^ cells in 200 µL of culture medium (0.5% FBS) was prepared and TYF (0, 100, 400 μg/mL) was added and mixed thoroughly. The culture medium was then added to the cell culture chambers and the chambers were incubated for 24 h. After incubation, the cells that had penetrated the cell culture chamber were fixed with 70% methanol for 10 min, followed by staining with Giemsa stain. Finally, using a phase-contrast microscope, cells that had migrated were randomly counted and analyzed in five different fields of view.

### Cell cycle analysis

TNBC cells were seeded (2.5 × 10^5^ cells/dish) in 6-cm culture dishes and allowed to adhere for 16–18 h under standard culture conditions. Cells were subsequently exposed to TYF at the indicated concentrations (MDA-MB-231: 0, 200, and 800 μg/mL; 4T1: 0, 100, and 400 μg/mL) and incubated for an additional 24 h. Following treatment, cells were harvested by trypsinization, washed with phosphate-buffered saline (PBS), and collected by centrifugation at 100 *g* for 15 min. Cell pellets were fixed by resuspension in 70% ethanol, followed by incubation at −20 °C for 30 min to achieve permeabilization. After fixation, cells were centrifuged again, ethanol was removed, and the pellets were resuspended in propidium iodide (PI) staining solution. Samples were incubated at 37 °C for 30 min in the dark prior to analysis. Cell cycle distribution was determined as previously described (Hua W‐J. et al., 2023).

### Apoptosis analysis

TNBC cells were seeded (2.5 × 10^5^ cells/dish) in 6-cm culture dishes and incubated for 18 h to allow cell attachment. Cells were then exposed to the indicated concentrations of TYF and returned to the incubator for an additional 48 h. Following treatment, cells were harvested by trypsinization, washed once with 1 mL of PBS, and collected by centrifugation at 100 *g* for 15 min. After removal of the supernatant, apoptotic cell populations were evaluated using the Alexa Fluor™ 488 Annexin V Apoptosis Kit (Thermo Fisher Scientific, United States) according to the manufacturer’s protocol. Apoptotic populations were quantified as previously described (Hua F. et al., 2023).

### Western blot assay

Cells were seeded (2.5 × 10^5^ cells/dish) per 6-cm culture dish and allowed to adhere prior to treatment. Cells were subsequently exposed to the indicated concentrations of TYF, and total protein was extracted after 3 h and 24 h of treatment. For protein extraction, concentrations and identification were followed previous study (Lin et al., 2019). Actin was used as the internal loading control. Detailed information regarding the antibodies used in this study is provided in Supplementary Table S1.

### Human receptor tyrosine kinase phosphorylation array

Two hundred µg of total protein from TYF-treated or control samples was applied to the Human Phospho-RTK Array (Catalog No.: AAH-PRTK-1-8; RayBiotech, Inc., GA, United States), following the manufacturer’s protocol and previous study (Hua W‐J. et al., 2023). After incubation and washing, phosphorylated tyrosine residues on activated RTKs were detected using a biotinylated anti-phosphotyrosine antibody, followed by horseradish peroxidase (HRP)-conjugated streptavidin. Signal development was carried out using a chemiluminescent substrate, and signals were captured for further analysis.

### Immunofluorescence assay

Cells were fixed with 4% paraformaldehyde in PBS, followed by permeabilization using 0.1% Triton X-100 for 5 min. Following permeabilization, non-specific binding sites were blocked by incubation in PBS containing 1% BSA at room temperature for 1 h. To visualize actin cytoskeletal organization, cells were stained with TRITC-conjugated phalloidin for 30 min using an actin cytoskeleton staining kit (Cat. No. FAK100; Sigma-Aldrich), according to the manufacturer’s instructions. Nuclear counterstaining was performed using 4′,6-diamidino-2-phenylindole (DAPI; Sigma-Aldrich). Fluorescence images were acquired using an ImageXpress Pico Automated Cell Imaging System (Molecular Devices, United States).

### Animal model

Six-week-old female BALB/c mice were obtained from the National Laboratory Animal Center (Taipei, Taiwan) and housed in the Animal Center of National Yang Ming Chiao Tung University (NYCU). All animal procedures were conducted in accordance with protocols approved by the Institutional Animal Care and Use Committee of NYCU (IACUC approval No. 1140410).

To establish a luciferase-expressing breast cancer model, 4T1 cells were transfected with a luciferase reporter construct (pLAS3w.FLuc.Ppuro, #C6-4-19; RNA Technology Platform and Gene Manipulation Core, Taiwan), generating the 4T1-Luc cell line. On day 0, 2 × 10^5^ 4T1-Luc cells were orthotopically injected into the mammary fat pads of female BALB/c mice.

Immunocompetent BALB/c mice were randomly assigned to two experimental groups (n = 5 per group): a control group receiving double-distilled water and a treatment group administered TYF at a dose of 2.4 g/kg/day. Treatments were delivered once daily by oral gavage for 14 consecutive days.

Body weight and tumor size were monitored throughout the experimental period. Tumor volume (V) was estimated using the standard formula: V = ½ × (length × width^2^), where length and width represent the longest and shortest tumor diameters, respectively. Tumor progression was further evaluated by *in vivo* bioluminescence imaging using a PhotonIMAGER Optima system (Biospace Lab, France). Bioluminescent signals were quantified as total photon flux (photons/second/cm^2^/steradian) for each animal.

### Statistical analysis

All data are presented as means ± standard deviation (SD). For comparisons among ≥3 groups, one-way ANOVA followed by an appropriate *post hoc* test was applied. For two-group comparisons, a two-tailed unpaired Student’s t-test was used via GraphPad Prism 8.0. P < 0.05 was considered statistically significant.

## Results

### The chemical profile of TYF

**FIGURE 1:**
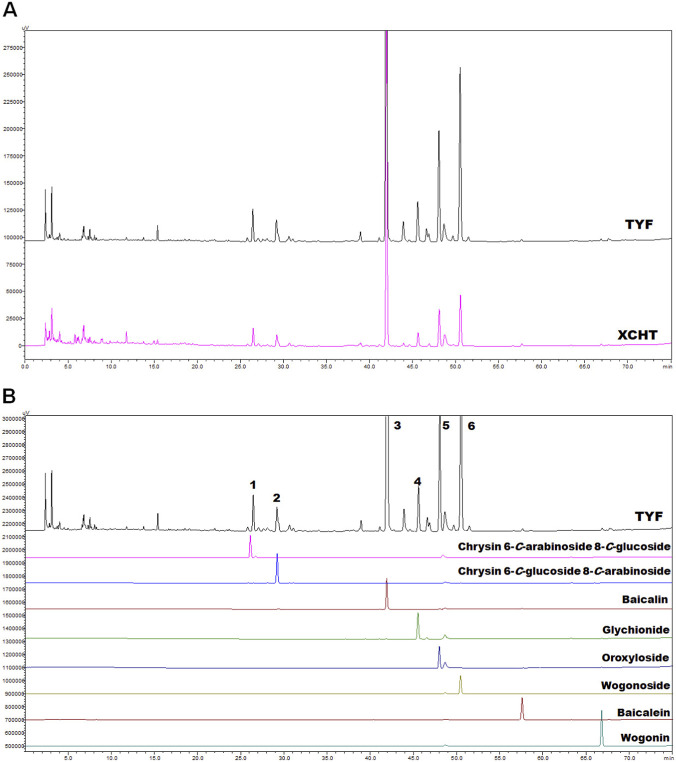
The fingerprint of TYF. **(A)** The fingerprints of TYF and XCHT. **(B)** Fingerprints of TYF and standard compounds from various herbal.

To investigate the chemical composition of TYF formula, derived from XCHT formula, a comparative analysis of the two formulas was conducted. The aqueous extracts (each of 10 mg) from TYF and XCHT were analyzed using reversed-phase high-performance liquid chromatography (RP-HPLC) equipped with a diode-array detector at the wavelength of 210 nm, as illustrated in Figure 1A. Although the HPLC fingerprints of TYF and XCHT appeared very similar, TYF exhibited several additional peaks, suggesting the presence of unique or enriched constituents.

Given the well-characterized phytochemical profiles of the constituent, reference standards were employed for the compound identification, including 8 flavonoids, chrysin 6-*C*-arabinoside 8-*C*-glucoside, chrysin 6-*C*-glucoside 8-*C*-arabinoside, baicalin, glychionide, oroxyloside, wogonoside, baicalein, wogonin. As shown in Figure 1B, six major peaks of HPLC fingerprint of TYF were identified, designated as peak 1 (t_*R*_ = 26.5 min), peak 2 (t_*R*_ = 29.5 min), peak 3 (t_*R*_ = 42.0 min), peak 4 (t_*R*_ = 46.0 min), peak 5 (t_*R*_ = 48.0 min), and peak 6 (t_*R*_ = 50.5 min). Based on retention times and comparison with the authentic standards, these peaks were assigned to chrysin 6-*C*-arabinoside 8-*C*-glucoside, chrysin 6-*C*-glucoside 8-*C*-arabinoside, baicalin, glychionide, oroxyloside, and wogonoside, respectively. The concentrations of these active compounds are listed in Supplementary Table S2.

### TYF inhibits the viability and colony formation of TNBC cells

**FIGURE 2:**
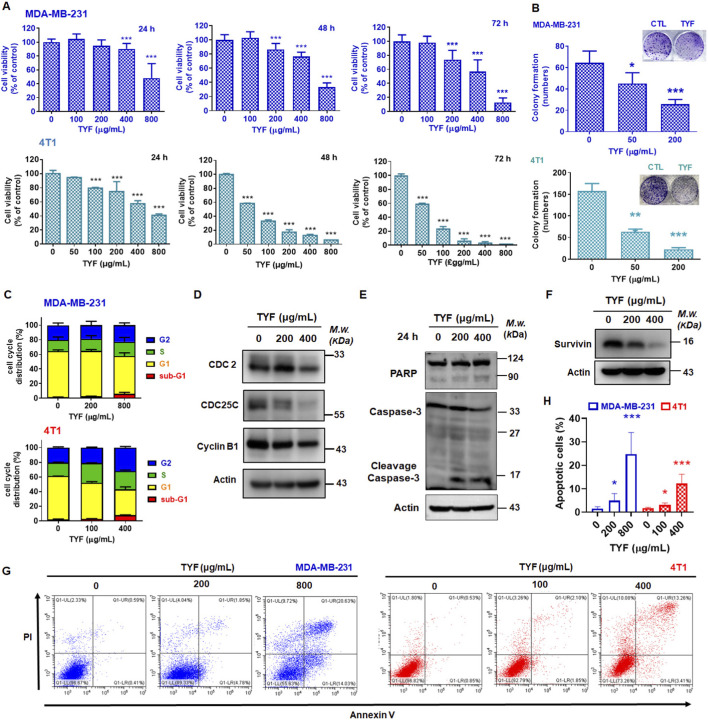
TYF inhibits cell viability of TNBC cells via induction of cell cycle arrest and apoptosis. **(A)** MDA-MB-231 and 4T1 cells were treated various concentrations (0–800 μg/mL) of TYF for 24, 48 and 72 h. Cell viability was measured by crystal violet assay. **(B)** Cells were treated with TYF (0, 50 and 200 μg/mL) for 7 days to analyze the colony formation. **(C–F)** Cells were treated with TYF (0, 100 and 400 μg/mL) for 24 h to analyze the cell cycle distribution **(C)** and markers **(D–F)**. Direct immunoblotting of the total protein extracts was performed using various indicated antibodies, and protein levels were examined through Western blot assay. The internal control was actin. **(G,H)** Cells were treated with various concentrations of TYF (0–800 μg/mL) for 48 h to analyze the apoptotic population. Significant differences were shown (*P < 0.05, compared with the untreated control group).

The inhibitory effects of TYF on TNBC cells were first evaluated *in vitro* using crystal violet-based viability and colony formation assays. As shown in Figure 2A, TYF treatment resulted in a marked reduction in cell viability in both MDA-MB-231 and 4T1 cells across the tested concentration range. Compared with untreated controls, TYF exposure led to progressively enhanced growth inhibition, with 4T1 cells exhibiting greater sensitivity than MDA-MB-231 cells. In MDA-MB-231 cells, treatment with a high concentration of TYF (800 μg/mL) produced inhibition rates of approximately 51%, 67%, and 90% following 24, 48, and 72 h of exposure, respectively. In contrast, 4T1 cells displayed pronounced growth suppression at lower TYF concentrations. When treated with 200 μg/mL TYF for 24, 48, and 72 h, inhibition rates reached approximately 59%, 87%, and 93.7%, respectively.

For comparison, the effects of the original XCHT decoction on TNBC cell viability were also examined. As shown in Supplementary Figure S1, XCHT treatment exerted minimal effects on MDA-MB-231 cells, whereas a moderate cytotoxic response was observed in 4T1 cells. Specifically, exposure of 4T1 cells to a high concentration of XCHT (800 μg/mL) resulted in inhibition rates of 0%, 58.7%, and 74.7% at 24, 48, and 72 h, respectively. Consistent with the viability data, colony formation assays demonstrated that TYF markedly impaired the clonogenic capacity of TNBC cells (Figure 2B). Treatment with TYF at 200 μg/mL reduced colony numbers by approximately 65% in MDA-MB-231 cells and 85% in 4T1 cells. Microscopic examination further revealed that TYF treatment decreased cell density and altered the characteristic spindle-shaped morphology of TNBC cells (Supplementary Figure S2).

Moreover, TYF showed no toxicity in normal breast epithelial MCF-10A and MCF-12A cells, with only a mild viability drop at 800 μg/mL (Supplementary Figure S3A, B). In normal fibroblast WI-38 cells, prolonged exposure caused a slight decrease in viability that remained modest even at the highest dose (Supplementary Figure S3C). The IC_50_ of TYF in these cells was shown in Table 1. TNBC cells were more sensitive to TYF compared to normal lung fibroblast cells. Although XCHT has an inhibitory effect on 4T1 cells, TYF exhibits a greater inhibitory effect on these breast cancer cells. These results confirmed that TYF suppressed cell viability of TNBC cells.

**TABLE 1:** IC_50_ of TYF in different cells.

IC_50_	MDA-MB-231 (μg/mL)	4T-1 (μg/mL)	MCF-10A (μg/mL)	MCF-12A (μg/mL)	WI-38 (μg/mL)
24 h	778.5 ± 38.52	545 ± 33.2	>800	>800	>800
48 h	667.2 ± 46.66	56.9 ± 0.37	>800	>800	>800
72 h	422.3 ± 36.41	42.6 ± 2.61	>800	>800	535.93 ± 111.30

### TYF increases cell cycle arrest and promotes apoptotic responses in TNBC cells

The potential mechanisms involved in anti-cancer effects of TYF on TNBC cells was assessed by initially examining cell cycle distribution in the TYF-treated cells. After treatment with TYF, the G2 phase and sub-G1 populations were increased compared to the control group (Figure 2C), suggesting that TYF may affect cell cycle checkpoint and apoptotic molecules. Next, we performed the Western blot assay to examine the expressions of the indicated markers. As shown in Figure 2D, TYF reduced CDC2, CDC25C, and Cyclin B1, which controlled G2 phase population (Dash and El-Deiry, 2005; Goldstone et al., 2001). TYF also increased cleavage PARP and caspase 3 (Figure 2E). These results indicated that TYF may trigger G2 phase arrest and consequently promote apoptosis. We thus performed propidium iodide (PI)/Annexin V-FITC double staining to analyze the apoptosis after 48 h of TYF treatment. As shown in Figures 2F–H, TYF significantly induced population of apoptotic cells but downregulated anti-apoptotic marker, survivin (Altieri, 2003). Together, these results confirmed that TYF exhibited anti-TNBC cells via induction of G2 phase arrest and apoptotic responses.

### TYF inhibits the mobility of MDA-MB-231 and 4T1 cells

**FIGURE 3:**
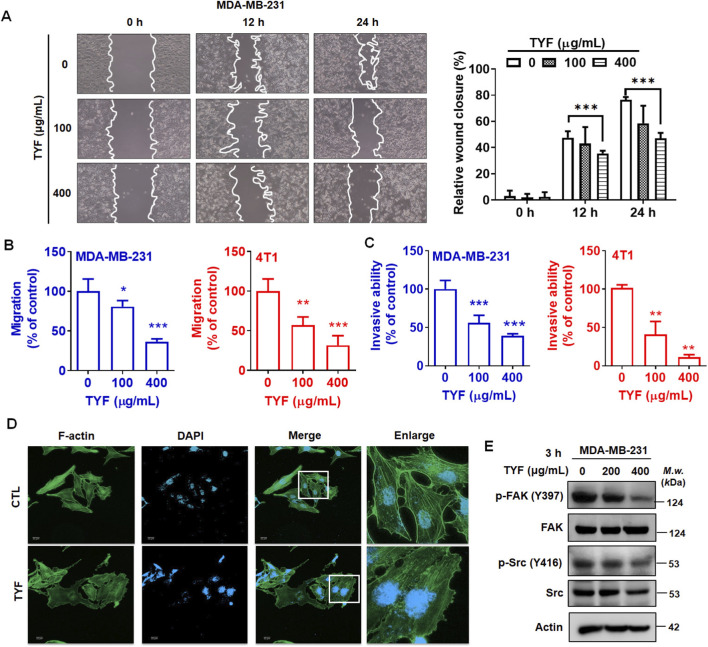
TYF inhibits the mobility of TNBC cells. **(A)** Wound closure assays in MDA-MB-231 cells. Cells were treated with TYF (100 and 400 μg/mL) and cultured at 0, 12 and 24 h. The data (% wound closure) are expressed as the percentage of the control (0 h) and are presented as the mean (±SD) of three separate determinations. **(B,C)** The migratory and invasive abilities were determined using Transwell assay. The migration **(B)** and invasion **(C)** of cells was inhibited by TYF (100 and 400 μg/mL) for 24 h. P values were considered significant at the level of 0.05* and were calculated using Student’s t-test (n = 3). **(D)** Immunofluorescence showing the actin-filament in MDA-MB-231 cells treated with TYF (100 μg/mL) for 24 h. The green color indicated F-actin stained with anti-phalloidin antibody and the blue color indicated nuclei stained with DAPI. Scale bar = 22 μm. **(E)** Total protein lysates derived from cells treated with various concentrations (0–400 μg/mL) of TYF for 3 h. Western blot analysis was performed for the determination of FAK/Src; actin was used as an internal protein-loading control.

TNBC is characterized by pronounced cellular heterogeneity and a high propensity for distant metastasis (Chen et al., 2025). To determine whether TYF affects migratory and invasive properties of TNBC cells, a series of *in vitro* motility assays were performed. Cell migration was first examined using a wound healing assay in MDA-MB-231 cells. As shown in Figure 3A, treatment with TYF at 400 μg/mL significantly inhibited wound closure by 40% after 24 h treatment. Consistent with this finding, Transwell® assays further demonstrated that TYF significantly suppressed both migratory and invasive capacities of TNBC cells, with reductions exceeding 50% (Figures 3B,C). Moreover, TYF disrupted the stress fiber of actin filament (Figure 3D). The phosphorylation of FAK (p-FAK) and Src (p-Src), which are involved in cell adhesion and migration (Dawson et al., 2021), was analyzed using Western blotting. Exposure of MDA-MB-231 cells to TYF for 3 h led to a notable reduction in phosphorylated FAK (p-FAK) and phosphorylated Src (p-Src) levels (Figure 3E). Collectively, these observations indicate that TYF interferes with key cellular processes governing TNBC cell motility.

### TYF inhibits the AXL and EGFR-regulated signaling pathways

**FIGURE 4:**
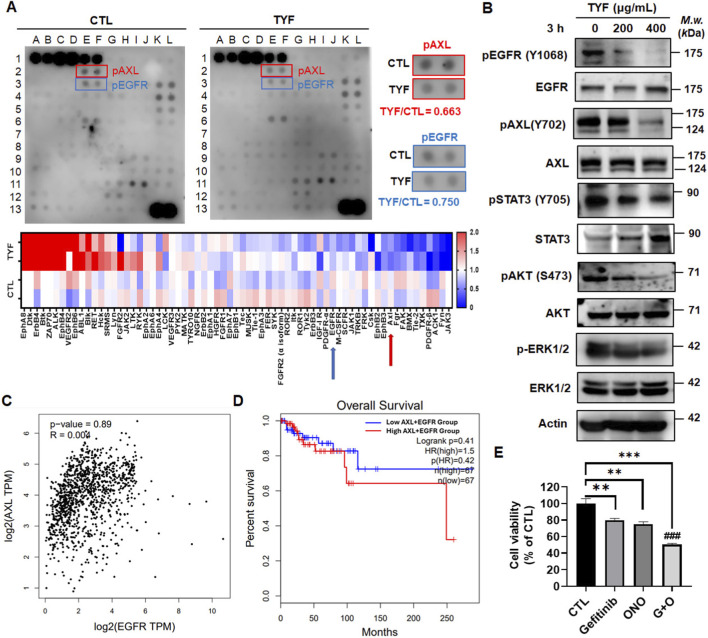
TYF inhibits the EGFR and AXL signaling of TNBC cells. **(A)** RTK array was performed after the cells received 3 h of treatment with TYF (100 μg/mL). The relative densities of phosphorylated EGFR and AXL are displayed. Lower panel: heat map showing changes in all phosphorylated RTKs. **(B)** Total protein lysates derived from cells treated with various concentrations (0–400 μg/mL) of TYF for 3 h. Western blot analysis was performed for the determination of indicated molecules; actin was used as an internal protein-loading control. **(C)** A correlation analysis was conducted using the GEPIA database to evaluate the relationship between AXL and EGFR transcript levels in TNBC samples. Gene expression was quantified in log2-transformed transcripts per million. **(D)** Overall survival analysis was conducted using the GEPIA database to explore the prognostic significance of concurrent AXL and EGFR expression in TNBC. **(E)** MDA-MB-231 cells were treated with gefitinib (10 μM), ONO-7475 (ONO; 10 μM), or a combination of both agents for 48 h. Cell viability was measured by crystal violet assay. The results are presented as means ± SD (***P < 0.001, compared with the untreated control group; ###P < 0.001, compared with the Gefitinib and ONO alone treatment group).

To explore signaling pathways potentially involved in the TYF-mediated suppression of TNBC cells, we employed a receptor tyrosine kinase (RTK) array (Supplementary Figure S4). As shown in Figure 4A, TYF treatment altered the phosphorylation status of multiple RTKs. Among these, AXL and EGFR displayed high basal phosphorylation levels in control cells, which were markedly reduced following TYF exposure. Based on these observations, downstream signaling events associated with EGFR and AXL activation were further examined. Western blot analyses demonstrated that TYF treatment attenuated EGFR phosphorylation and was accompanied by reduced activation of key downstream effectors, including STAT3, AKT, and ERK1/2 (Figure 4B). In parallel, TYF also decreased the phosphorylation level of AXL, indicating coordinated modulation of these two receptor tyrosine kinases. Complementary big data analysis of TNBC patient samples further revealed a positive correlation between EGFR and AXL expression (Figure 4C). Moreover, patients exhibiting high co-expression of EGFR and AXL had significantly poorer overall survival (Figure 4D). We therefore further investigated the effects of EGFR and AXL inhibition on viability of TNBC cells. Notably, while individual inhibition of EGFR or AXL reduced cell viability, the combined inhibition of both receptors produced an enhanced suppressive effect (Figure 4E). The combined administration increased the subG1 population and induced a slight apoptotic response (Supplementary Figure S5). These results demonstrate that TYF can simultaneously inhibit EGFR and AXL-mediated pathways to suppress TNBC viability.

### TYF inhibits tumor progression of 4T1 cells-bearing BALB/c mouse

**FIGURE 5:**
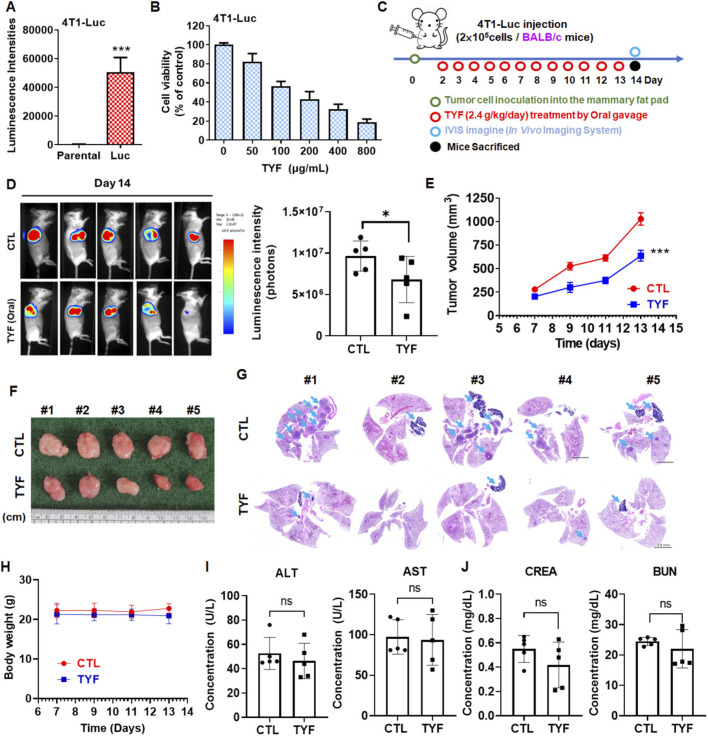
TYF suppresses tumor growth of 4T1-bearing BALB/c mice. **(A)** Luminescence intensity of 4T1-Luc cells was determined. **(B)** 4T1-Luc cells were treated with various concentrations of TYF for 48 h. Cell viability was measured by crystal violet assay. **(C)** Scheme of animal experiment design. **(D)** The growth of tumor as monitored using bioluminescence imaging on day 14. Right panel: corresponding quantification of bioluminescence imaging signals (luminescent intensity of photons). **(E)** The tumor volume was monitored on the indicated days. Each group contained five mice, and the results are presented as means ± SD (***P < 0.001). **(F)** Representative tumor images, captured on the 14th day of sacrifice, were presented. **(G)** Hematoxylin and eosin-stained lung sections (arrows indicate tumor nodules). **(H)** Body weights of the mice were recorded. **(I,J)** Serum biochemical parameters in the normal, control, and TYF-treated groups are shown (n = 5 per group). **(I)** Aspartate aminotransferase (AST) and alanine aminotransferase (ALT) levels were measured to assess liver toxicity. **(J)** Blood urea nitrogen (BUN) and creatinine levels were evaluated to determine kidney toxicity.

The *in vivo* effects of TYF on breast tumor progression were evaluated using a luciferase-expressing 4T1 (4T1-Luc) orthotopic mouse model combined with bioluminescence imaging. Stable luciferase expression in 4T1-Luc cells was confirmed by the presence of robust bioluminescent signals (Figure 5A). *In vitro* viability assays demonstrated that TYF reduced the viability of 4T1-Luc cells in a concentration-dependent manner following 48 h of treatment, with an estimated IC_50_ of approximately 150 μg/mL (Figure 5B). For *in vivo* assessment, 4T1-Luc cells were orthotopically implanted into the mammary fat pads of female mice, followed by daily oral administration of TYF according to the experimental schedule (Figure 5C). Longitudinal bioluminescence imaging revealed that mice receiving TYF exhibited markedly lower luminescent signals compared with control animals, indicating reduced tumor burden (Figure 5D). Quantitative analysis of bioluminescence intensity, together with gross tumor imaging, further demonstrated that TYF treatment significantly suppressed tumor growth, as reflected by decreased tumor volumes relative to the control group (Figures 5E,F). Moreover, the extent of pulmonary metastasis was evaluated. TYF-treated mice developed fewer metastatic nodules compared with control animals (Figure 5G). Throughout the treatment period, no significant changes in body weight were observed between groups (Figure 5H). Moreover, biochemical assessments revealed no apparent liver or kidney toxicity following TYF administration (Figures 5I,J), suggesting favorable tolerability under the experimental conditions.

## Discussion

XCHT has been widely prescribed to alleviate emotional distress and psychosomatic discomfort in patients with breast cancer and is commonly regarded as an adjunctive therapy that complements conventional treatments (Deng et al., 2021; Liang et al., 2020). In clinical practice, XCHT is not primarily prescribed with the intention of directly suppressing tumor cell proliferation, but rather to regulate emotional imbalance and systemic dysfunction associated with chronic disease states. Consistent with this traditional therapeutic intent, our *in vitro* analyses showed that XCHT exhibited minimal direct cytotoxic activity against TNBC cells. In this study, we hypothesized that the sugar-rich ingredients of XCHT might introduce metabolic confounding factors when evaluating its biological relevance in modern experimental models of TNBC. Importantly, this refinement was not intended to negate the traditional role of ZhiGancao and Dazao, but rather to facilitate a clearer ethnopharmacological evaluation of XCHT-related core components under contemporary disease and metabolic conditions. For example, Dazao exhibits partially overlapping supportive functions with Dangshen, while ZhiGancao primarily serves a harmonizing role in TCM theory and is not considered a principal contributor to the core pharmacological activity of the formula. Supporting this rationale, our preliminary analyses indicated that the glucose content of XCHT was approximately 2.5-fold higher than that of TYF (data not shown). Accumulating evidence indicates that increased glucose availability is associated with accelerated proliferation, enhanced migration and invasion, epithelial-mesenchymal transition, and treatment resistance in TNBC cells (Lei et al., 2023; Niu et al., 2025). We found that glucose supplementation partially restored viability under TYF treatment (Supplementary Figure S6). Therefore, modulation of glucose content may represent an additional metabolic context influencing the observed anti-tumor effects of TYF. Moreover, elevated sugar levels may attenuate the observable biological effects of the original formulation in anticancer-related assays.

Using this refined formulation as an ethnopharmacological probe, we observed that TYF selectively modulated TNBC-associated cellular behaviors in both *in vitro* and *in vivo* models. TYF reduced TNBC cell viability while exerting minimal cytotoxicity toward non-malignant breast epithelial cells and human fibroblasts, indicating favorable selectivity toward malignant phenotypes. In addition, TYF induced G2 phase cell cycle arrest and apoptotic responses and significantly impaired TNBC cell migration and invasion. These effects were accompanied by disruption of actin cytoskeletal organization and suppression of key motility-related signaling molecules, including FAK and Src (Dawson et al., 2021; Lo et al., 2023), suggesting that TYF may influence biological processes relevant to tumor dissemination rather than exerting nonspecific cytotoxicity.

At the signaling level, RTK profiling revealed that TYF markedly reduced phosphorylation of EGFR and AXL, two kinases frequently overexpressed in TNBC and associated with aggressive disease phenotypes and poor clinical outcomes (Masuda et al., 2012; Ozyurt and Ozpolat, 2023). Analysis of public datasets further demonstrated that co-overexpression of EGFR and AXL correlates with reduced overall survival in TNBC patients (Figure 4C). Previous studies have reported functional interactions between EGFR and AXL, which converge on downstream PI3K/Akt and MAPK pathways and contribute to resistance against single-agent targeted therapies (Heideman and Hynes, 2013; Wu et al., 2025). Within this context, the concurrent modulation of EGFR- and AXL-associated signaling observed in the present study should be interpreted as a system-level effect characteristic of multi-component herbal prescriptions, rather than as evidence of a single-target pharmacological intervention. Similar dual inhibition strategies have been shown to suppress tumor growth and overcome resistance in preclinical models (Kim, 2025), further supporting the biological relevance of coordinated pathway regulation. Interestingly, although TYF markedly reduced phosphorylation of EGFR and AXL, we observed a modest increase in phosphorylation of several other RTKs. This differential regulation reflects TYF may induce network-level signaling rewiring, and that increases in certain RTKs may reflect compensatory responses or redistribution of signaling upon suppression of dominant nodes.

This study focused on the formulation-level effects of TYF rather than single-herb interventions, with the aim of improving translational relevance while preserving the holistic principles of traditional Chinese medicine. TYF was rationally reformulated by rebalancing five core herbal components to enhance “heat-clearing and detoxifying” properties in accordance with TCM theory, while retaining stress- and emotion-regulating functions that may support treatment adherence. Chemical fingerprint analysis identified six characteristic peaks, including baicalin, wogonoside, and oroxyloside, which are consistent with major flavonoids previously reported in XCHT (Du et al., 2018). These flavonoids may collectively contribute to the observed TNBC-suppressive effects. For example, baicalin has been extensively reported to inhibit invasive breast cancer phenotypes through suppression of proliferation, migration, invasion, and epithelial-mesenchymal transition (EMT) (Hua F. et al., 2023). Consistent with these observations, TYF exhibited comparable anti-tumor activities, although overt induction of epithelial markers was not observed (data not shown). Wogonoside has demonstrated anti-metastatic and anti-angiogenic effects in breast cancer models (Huang et al., 2016), while oroxyloside is associated with inhibition of glycolytic metabolism and suppression of EMT characteristic of TNBC (Sun et al., 2019). Overall, TYF contains multiple flavonoid clusters that may collectively contribute to network-level modulation of TNBC-relevant pathways, consistent with the multi-component nature of TCM prescriptions. Further analysis of TYF using ultra-performance liquid chromatography coupled with tandem mass spectrometry and systematic network pharmacology would be more valuable for this study. Prior studies showed the network pharmacology of XCHT in liver injury and thyroid cancer, demonstrating that XCHT can induce apoptosis by affecting the PI3K/AKT pathway (Hong et al., 2017; Wang et al., 2022), consistent with our findings. Furthermore, we demonstrate that TYF reduces phosphorylation of EGFR and AXL, and our unpublished data show that baicalin has the potential to bind to the kinase domains of EGFR and AXL (data not shown), which may provide evidence of why XCHT affects this pathway.

Although these findings underscore the therapeutic potential of TYF, several important limitations warrant consideration. The specific phytochemical clusters responsible for modulation of EGFR- and AXL-associated signaling were not delineated. Detailed plasma pharmacokinetic and metabolic analyses were beyond the scope of this work. Previous *in vivo* studies of XCHT have reported that key flavonoids, including baicalin and wogonoside, are detectable in mouse circulation following oral administration, and metabolic profiling further supports their systemic presence together with related conjugated metabolites (Sun et al., 2015; Du et al., 2019). Consistently, our fingerprint analysis identified baicalin and wogonoside among the major peaks, and quantitative profiling revealed that baicalin is enriched in TYF compared with XCHT (92.71 vs. 37.16 mg/g), supporting the potential contribution of these compounds to the observed anti-TNBC activity. It is still important to emphasize that traditional herbal prescriptions are intentionally designed to achieve therapeutic efficacy through synergistic and buffering interactions among multiple components rather than through isolated compounds. Accordingly, this study prioritized validation of the ethnopharmacological relevance of a XCHT-derived decoction at the formulation level, supported by chemical fingerprinting to ensure reproducibility and consistency. Moreover, while removal of ZhiGancao and Dazao facilitated mechanistic interpretation under experimental conditions, further studies are needed to investigate how metabolic context and dietary factors interact with traditional formulations in clinical settings. Finally, the present *in vivo* experiments were conducted using a syngeneic murine 4T-1 model in immunocompetent mice. Validation in human TNBC xenograft models established in immunodeficient mice will be important to further assess dose responsiveness and translational relevance in human-derived tumors. Moreover, studies incorporating subchronic safety analysis and host-microbiome interaction assessment will further elucidate the systemic impact of TYF.

It is important to emphasize that traditional herbal prescriptions are intentionally designed to achieve therapeutic efficacy through synergistic and buffering interactions among multiple components rather than through isolated compounds. Accordingly, this study prioritized validation of the ethnopharmacological relevance of a XCHT-derived decoction at the formulation level, supported by chemical fingerprinting to ensure reproducibility and consistency. Moreover, while removal of ZhiGancao and Dazao facilitated mechanistic interpretation under experimental conditions, further studies are needed to investigate how metabolic context and dietary factors interact with traditional formulations in clinical settings. Finally, the present *in vivo* experiments were conducted using a syngeneic murine 4T1 model in immunocompetent mice. Validation in human TNBC xenograft models established in immunodeficient mice will be important to further assess dose responsiveness and translational relevance in human-derived tumors.

## Conclusion

This study provides ethnopharmacologically grounded and mechanistic evidence supporting the relevance of an XCHT-derived decoction (TYF) in TNBC models. The observed modulation of EGFR/AXL-associated signaling and metastatic phenotypes offers biological insight into the contemporary adjunctive use of XCHT-related prescriptions in breast cancer–associated complex disease states.

## Data Availability

The original contributions presented in the study are included in the article/Supplementary Material, further inquiries can be directed to the corresponding author.
