# Supplementary material for: Xiao Chai Hu Tang-derived decoction (Tung-Yi Fang) suppresses triple negative breast cancer cells in vitro and in vivo via regulating EGFR/AXL-mediated signaling
Source: Front Pharmacol. 2026 Mar 13;17:1778030. doi: 10.3389/fphar.2026.1778030 (PMC13021619; doi:10.3389/fphar.2026.1778030)
Supplement: Supplementary file 1 [file DataSheet1.pdf]

Xiao Chai Hu Tang-derived decoction (Tung-Yi Fang) suppresses triple negative breast cancer cells *in vitro* and *in vivo* via regulating EGFR/AXL-mediated signaling

Li-Lan Liao, Zhi-Hu Lin, Chia-Ching Liaw, Hsin Yeh,Wei-Hao Wang,
Yun-Chih Chen, Yi-An Lin, Ai-Jung Tseng, Yu-Chun Lin, Wen-Hsin Tsai,
Chi-Hong Chao, Mei-Kuang Lu, Chung-Hua Hsu, Tung-Yi Lin

Supplementary Information

Supplementary Table

Table S1. The information of indicated antibodies.

Antibody	*Company*	*Cat.No.*	*Dilution*
**p-EGFR (Tyr1068)**	GeneTex	GTX132810	1:1000
**p-AXL (Tyr702)**	Cell singal	# 5724	1:1000
**p-FAK (Tyr 397)**	Santa Cruz	SC 81493	1:1000
**p-STAT3 (Tyr705)**	GeneTex	GTX118000	1:2000
**p-SRC (Tyr416)**	GeneTex	GTX24816	1:1000
**p-AKT (Ser 473)**	Cell singal	# 4060	1:2000
**p-ERK (Thr202/Tyr204)**	Cell singal	# 9101S	1:1000
**EGFR**	GeneTex	GTX121919	1:2000
**AXL**	GeneTex	GTX101345	1:1000
**FAK**	Cell singal	#3285	1:1000
**STAT3**	GeneTex	GTX104616	1:2000
**SRC**	Bio source	44-6582B	1:1000
**AKT**	Ireal	IR171666	1:2000
**ERK**	Ireal	IR181705	1:2000
**Cyclin B1**	GeneTex	GTX100911	1:1000
**CDC25C**	Santa Cruz	SC13181	1:1000
**Survivin**	GeneTex	GTX100441	1:1000
**PARP**	Cell singal	# 9542S	1:1000
**Caspase 3**	Cell singal	# 9662	1:1000
**Actin**	GeneTex	GTX109639	1:10000

Table S2. The quantitative analysis of compounds **1-6** in TYF and XCHT.

Compound		Calibration curve	mg/g	
			TYF	XCHT
**1**	Chrysin 6-*C*-arabinoside 8-*C*-glucoside	y = 20943x + 10105	8.06	2.41
**2**	Chrysin 6-*C*-glucoside 8-*C*-arabinoside	y = 25590x + 9968	7.74	3.89
**3**	Baicalin	y = 78136x - 349717	92.71	37.16
**4**	Glychionide A	y = 79630x + 16011	6.07	2.33
**5**	Oroxyloside	y = 30129x + 39006	10.28	3.35
**6**	Wogonoside	y = 77409x + 752	21.29	6.70

Supplementary Figures

Supplementary Figure 1

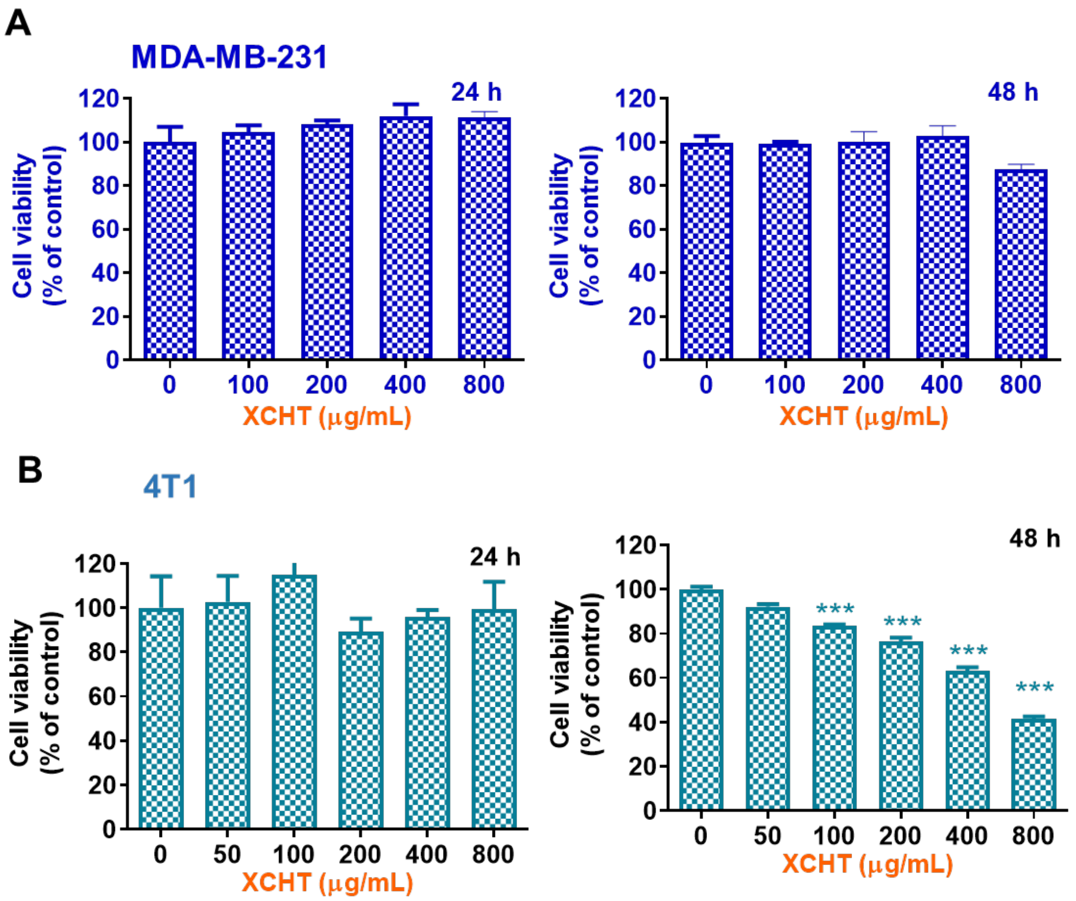

Supplementary Figure 1. XCHT does not affect cell viability of TNBC cells.

MDA-MB-231 (**A**) and 4T1 (**B**) cells were treated various concentrations (0-800 μg/mL) of XCHT for 24 and 48 h. Cell viability was measured by crystal violet assay. Data are presented as mean ± SD from independent experiments. Significant differences were shown (* $p < 0.05$, compared with the untreated control group).

Supplementary Figure 2

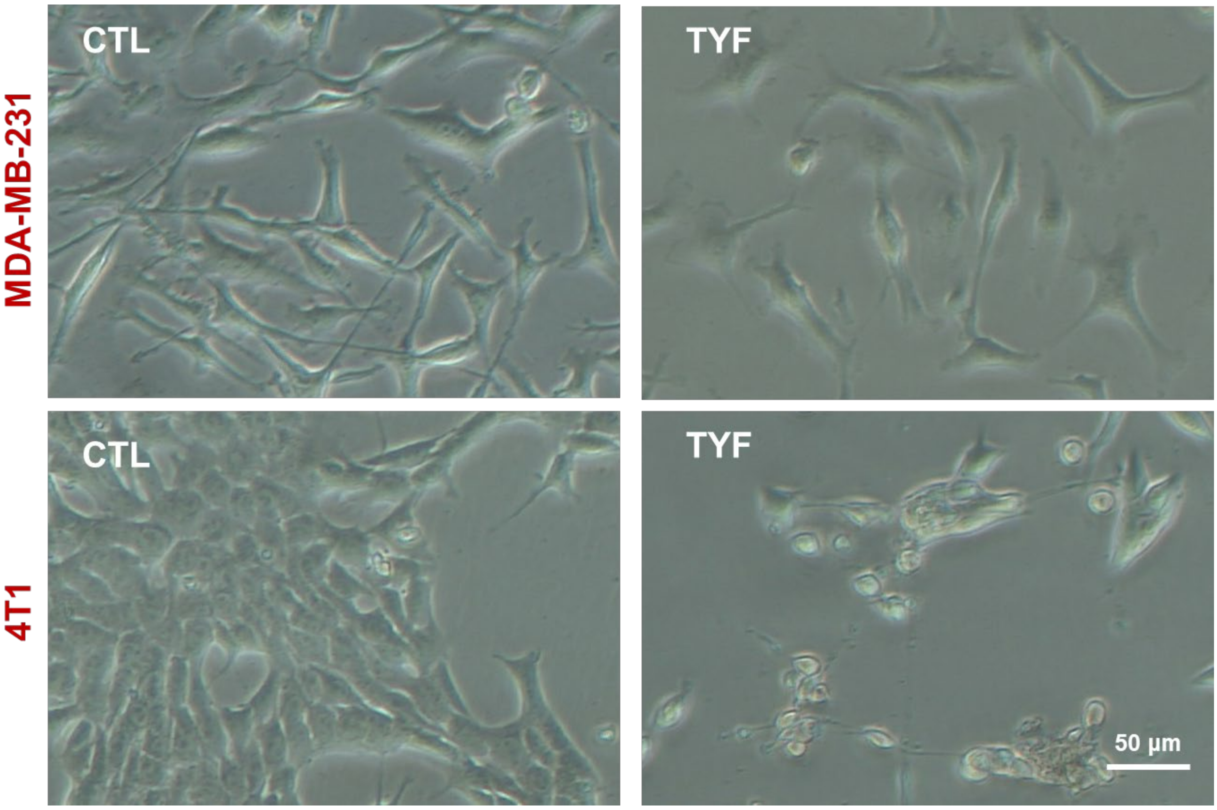

Supplementary Figure 2. TYF reduces cell number and induces changes in cell morphology. MDA-MB-231 and 4T1 cells were treated TYF (400 μg/mL) for 24 h. Images were captured using microscope. Scale bar = 50 μm.

Supplementary Figure 3

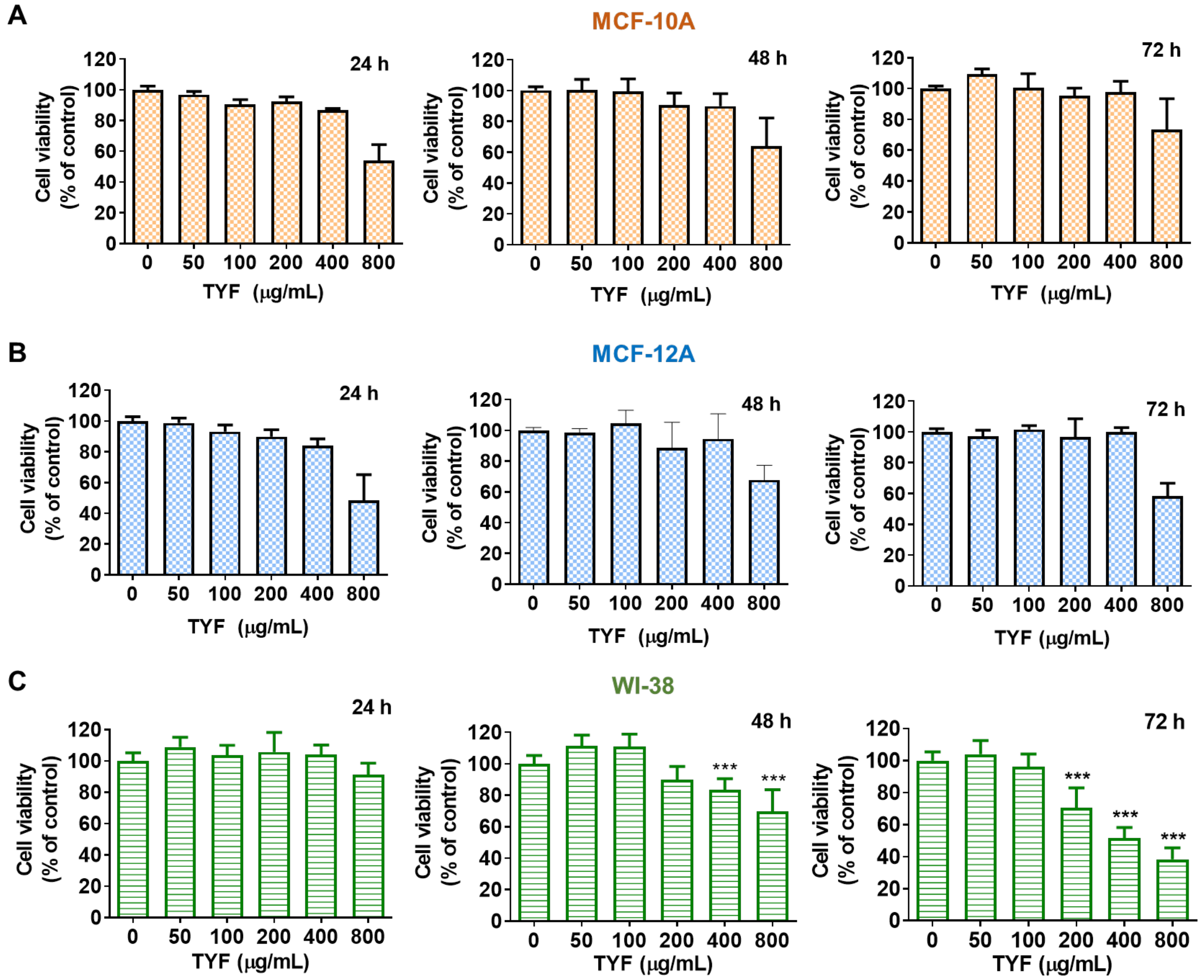

Supplementary Figure 3. TYF does not affect cell viability of immortalized epithelial and fibroblast cells.

Breast epithelial MCF-10A (**A**), MCF-12A (**B**) and lung fibroblast WI-38 (**C**) cells were treated various concentrations (0-800 μg/mL) of TYF for 24, 48 and 72 h. Cell viability was measured by crystal violet assay. Data are presented as mean ± SD from independent experiments. Significant differences were shown (*** $p < 0.001$, compared with the untreated control group).

Supplementary Figure 4

	A	B	C	D	E	F	G	H	I	J	K	L
1	POS 1	POS 1	POS 2	POS 2	POS3	POS3	ABL1	ABL1	ACK1	ACK1	ALK	ALK
2	NEG	NEG	NEG	NEG	Axl	Axl	Blk	Blk	BMX	BMX	Btk	Btk
3	Csk	Csk	Dtk	Dtk	EGFR	EGFR	EphA1	EphA1	EphA2	EphA2	EphA3	EphA3
4	EphA4	EphA4	EphA5	EphA5	EphA6	EphA6	EphA7	EphA7	EphA8	EphA8	EphB1	EphB1
5	EphB2	EphB2	EphB3	EphB3	EphB4	EphB4	EphB6	EphB6	ErbB2	ErbB2	ErbB3	ErbB3
6	ErbB4	ErbB4	FAK	FAK	FER	FER	FGFR1	FGFR1	FGFR2	FGFR2	FGFR2 (α isoform)	FGFR2 (α isoform)
7	Fgr	Fgr	FRK	FRK	Fyn	Fyn	Hck	Hck	HGFR	HGFR	IGF-I R	IGF-I R
8	Insulin R	Insulin R	Itk	Itk	JAK1	JAK1	JAK2	JAK2	JAK3	JAK3	LCK	LCK
9	LTK	LTK	Lyn	Lyn	MATK	MATK	M-CSFR	M-CSFR	MUSK	MUSK	NGFR	NGFR
10	PDGFR-α	PDGFR-α	PDGFR-β	PDGFR-β	PYK2	PYK2	RET	RET	ROR1	ROR1	ROR2	ROR2
11	ROS	ROS	RYK	RYK	SCFR	SCFR	SRMS	SRMS	SYK	SYK	Tec	Tec
12	Tie-1	Tie-1	Tie-2	Tie-2	TNK1	TNK1	TRKB	TRKB	TXK	TXK	NEG	NEG
13	Tyk2	Tyk2	TYRO10	TYRO10	VEGFR2	VEGFR2	VEGFR3	VEGFR3	ZAP70	ZAP70	POS2	POS2

Supplementary Figure 4. Human phospho-RTK array coordinates.

Supplementary Figure 5

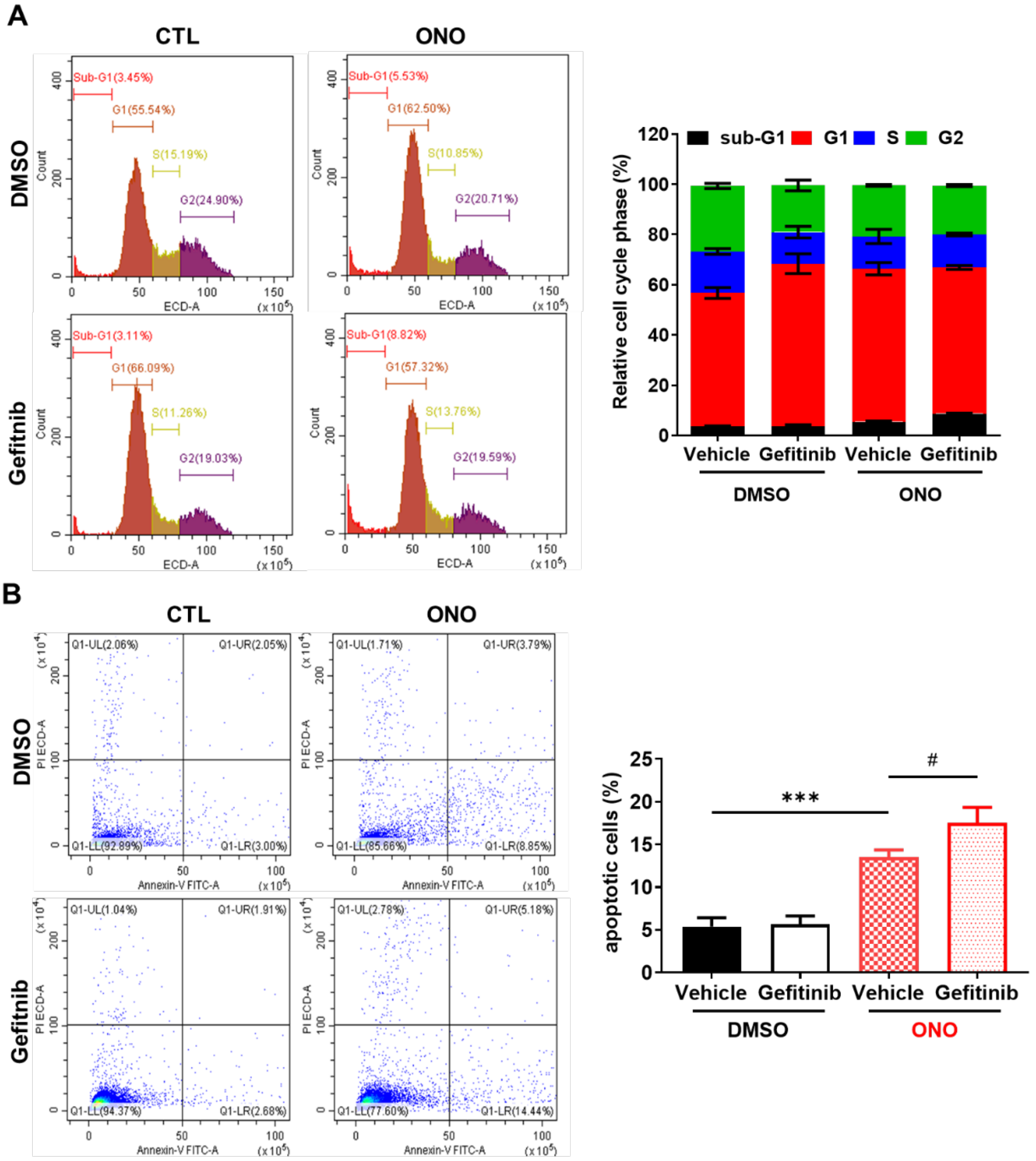

Supplementary Figure 5. Effects of gefitinib and ONO-7475 on cell cycle distribution and apoptosis in MDA-MB-231 cells. Cells were treated with gefitinib (10 µM), ONO-7475 (ONO; 10 µM), or the combination of both agents for 24 h. (A) Cell cycle distribution was analyzed by flow cytometry. Representative histograms (left) and quantitative analysis (right) show the percentage of cells in sub-G1, G1, S, and G2/M phases under each treatment condition. (B) Apoptosis was assessed by Annexin V-FITC/PI double staining and flow cytometric analysis. Representative dot plots (left) and quantification (right) illustrate the proportions of viable cells, early apoptotic cells, late apoptotic cells, and necrotic cells. Data are presented as mean ± SD from independent experiments. Significant differences were shown ($^{\#} < p$ 0.05, *** $p < 0.001$).

Supplementary Figure 6

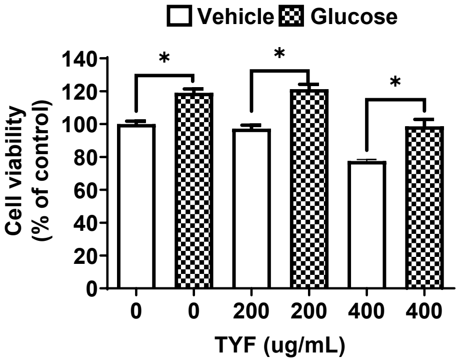

Supplementary Figure 6. Glucose abolishes TYF-inhibited cell viability of MDA-MB-231 cells. Cells were treated with TYF (0-400 μg/mL) and glucose (90 μg/mL) for 24 h. Cell viability was measured by crystal violet assay. Significant differences were shown (* $p < 0.05$, compared with the control group).
